# The Impact of *Origanum vulgare* Supplementation on Human Asthenozoospermic Sperm Parameter Quality

**DOI:** 10.1155/2023/8093795

**Published:** 2023-06-24

**Authors:** Ahlam Zarhouti, Moudou M. Mbaye, Boutaina Addoum, Noureddine Louanjli, Bouchra El Khalfi, Abdelaziz Soukri

**Affiliations:** ^1^Laboratory of Physiopathology, Molecular Genetics & Biotechnology, Faculty of Sciences Ain Chock, Health and Biotechnology Research Centre, Hassan II University of Casablanca, Maarif B.P 5366, Casablanca, Morocco; ^2^Laboratory of Medical Analysis, Andrology, LABOMAC, Casablanca, Morocco; ^3^IRIFIV In Vitro Fertilization Centre, IRIS Clinic, Casablanca, Morocco

## Abstract

Male infertility is a complex multifactorial disease and a real health problem; 50% of infertile men have identifiable causes detectable by fundamental sperm analysis. Numerous research studies have shown the possibility of treating abnormal semen samples with some drugs before artificial insemination, yet evidence of the drug's effectiveness remains minimal. In our previous work, we tested the effectiveness of some essential oils, such as eucalyptus (*Eucalyptus globulus* Labill.), oregano (*Origanum vulgare* L.), and sage (*Salvia officinalis* L.) on sperm parameters. The essential oil of oregano showed the best ameliorative effect. In present, we examined the effect of the essential oil of *O. vulgare* on the physiological parameters and the specific activity of certain antioxidant enzymes such as catalase (CAT), superoxide dismutase (SOD), lipid peroxidation rate (MDA), and GAPDH and antioxidant and metabolic biomarkers, characterizing the quality of human sperm. The results showed that *in vitro* supplementation of oregano significantly improves the mobility and antioxidant activities, without harmful effects on the integrity of the sperm's DNA, and that the selected concentration of oregano EO is nontoxic and may be considered a therapeutic alternative to heal sperm motility problems in asthenozoospermic patients.

## 1. Introduction

Infertility is a disease that affects the reproductive systems of both men and women and is characterized by the inability to conceive a child after having frequent, unprotected sex for at least a year [1]. The male component is the main contributing factor in about 50% of infertility cases [2], affecting 8–12% of couples worldwide [3]. The causes of infertility in men are diverse and varied, including sexual problems, hormonal disorders, psychological problems, physical problems, lifestyle problems, and chromosomal abnormalities [3].

The primary technique used to assess male fertility is semen analysis [4]. In addition to assessing semen quality, the semen analysis can be used to screen patients for in vitro fertilization (IVF) or intracytoplasmic sperm injection treatment (ICSI), since the latter requires a sperm sample with a concentration of 10 × 10^6^ sperm/mL of which at least 30% are mobile and 15% have progressive motility [5]. Indeed, several studies have shown that reactive oxygen species (ROS) play an essential role in the success of IVF [6]. Their abundance in sperm and seminal plasma is in negative correlation with IVF/ICSI fertilization, embryo quality, and live-birth rates [7].

On the other hand, abnormal sperm parameters, for example, low sperm concentration, poor motility, and poor morphology, have positive correlations with an increase in the level of reactive oxygen species in the sperm [8]. Nonetheless, to support healthy reproductive processes such as sperm capacitation hyperactivation and acrosome response, ROS are essential at normal physiological levels [9]. Therefore, a balance between ROS and the antioxidant system is essential for the proper performance of sperm and successful fertilization [10].

To treat sperm quality problems before IVF, our laboratory carried out a series of studies, mainly devoted to test the effects of sage (*S. officinalis*), oregano (*O. vulgare*), and eucalyptus (*E. globulus*) on the improvement of spermatic mobility and vitality.

For a better understanding of the action mechanism behind the ameliorative effect of these oils, especially their control of oxidative stress, we initially studied the main role of oregano supplementation on physiological parameters (mobility and vitality) and sperm DNA quality (DNA fragmentation index). Then, we measured some antioxidant and metabolic biomarkers (SOD, catalase, MDA, and GAPDH).

## 2. Materials and Methods

### 2.1. Plant Material

The essential oil of *O. vulgare* was supplied by the Laboratory of Physiopathology, Genetic Molecular, and Biotechnology, Ain Chock Faculty of Sciences, Morocco.

### 2.2. Data Collection

This study was conducted following ethical guidelines, and informed consent was obtained from all recruited individuals. The samples were collected at the Laboratory of Medical Analysis and Reproductive Biology, “Labomac,” Casablanca, Morocco.

Sperm samples are obtained by masturbation after 3-4 days of abstinence. They were then liquefied at 37°C under 5% CO_2_ until they were used. All samples were prepared and analyzed according to the current World Health Organization (WHO) guidelines and standards.

The study was divided into two groups: Group A (*n* = 12) consisted of samples from men classified as normozoospermic (20 × 10^6^/mL concentration, 32% progressive motility) and Group B (*n* = 12) composed of patients who are classified as asthenozoospermic (20 × 10^6^/mL concentration, the progressive motility is <32%).

### 2.3. Preparation of Samples

After 30 minutes of ejaculate collection, the samples of asthenozoospermic sperm were divided into two equal aliquots in 1.5 mL Eppendorf tubes: the first tube was identified as a control and the second was incubated with oregano oil at a final dilution of 10^−2^. Four incubations were performed for (5, 10, and 30 at 60 min) at 37°C below 5% CO_2_.

### 2.4. Analysis of the Spermatic Parameters

#### 2.4.1. Analysis of Mobility

The effect of oregano oil on the advanced parameters of human sperm mobility was evaluated at different incubation intervals: (*t* = 0 min), 10 min later (*t* = 10 min), and (*t* = 30 min), and the impact of oregano oil on sperm mobility was manually evaluated according to the 2021 WHO recommendations.

#### 2.4.2. Analysis of Vitality

Sperm vitality was assessed using the WHO values. To achieve our objective, we used a 2% eosin staining immediately after stabilization. The slides were examined under an optical microscope.

#### 2.4.3. Analysis of DNA Fragmentation

The integrity of the spermatic DNA was assessed by the TUNEL test using a commercial kit (Roche Diagnostics, Lewes, UK).

### 2.5. Enzymatic Bioassays

#### 2.5.1. Separation of Spermatozoa and Lysis

The sperm was separated from seminal plasma by centrifugation (600*g* for 10 min), the sperm pellet was recovered, and a sperm lysate in distilled water was prepared for biochemical analysis.

#### 2.5.2. Determination of CAT Activity

The activity of catalase was determined by the Aebi method [11].

Following the consumption of peroxide hydrogen at 240 nm, catalase activity was measured. The reaction mixture contained 7.5 mM H_2_O_2_ in 50 mM potassium phosphate buffer (pH 7.0) and 50 *μ*L enzyme extract. CAT activity was calculated using the molar extinction coefficient of H_2_O_2_ (0.0394 mM^−1^ cm^−1^) and expressed as *μ*mol H_2_O_2_ consumption/min/mg of protein.

#### 2.5.3. Determination of SOD Activity

The technique of measuring the SOD activity was identified in 1986 by Paoletti and his collaborators [12].

This method is based on the inhibition of NADH oxidation by superoxide dismutase. The decrease in the oxidation rate of NADH is proportional to the concentration of the enzyme. The measurement of activity is determined by a spectrophotometer at 340 nm. The molar extinction coefficient of the NADH is 6220 M^−1^ cm^−1^. Enzyme activity is expressed in SOD unit/min/mg protein.

#### 2.5.4. Determination of GAPDH Activity

The enzyme activity of NAD + dependent phosphorylating GAPDH is determined by using a spectrophotometer at 30°C by measuring the occurrence of NADH at 340 nm [13]. The enzyme preparation is added to the reaction mixture that contains tricine-NaOH buffer (50 mM, pH 8), sodium arsenate 10 mM, NAD+ 0.4 mM, and D-G3P 0.4 mM. The total volume of the reaction mixture is 1 mL. The molar extinction coefficient of NADH is 6220 M^−1^ cm^−1^. Enzyme activity is expressed in units of activity/mg protein.

#### 2.5.5. Determination of MDA Activity

The level of lipid peroxidation is quantified in terms of substances reacting with thiobarbituric acid (TBARS) according to the Samokyszyn and Marnet method [14]. 100 *μ*L of the crude extract is added to 900 *μ*L of a solution consisting of 0.375% thiobarbituric acid and 15% trichloroacetic acid. The mixture is then placed in a 100°C waterbath for 15 minutes and cooled in the ice to stop the reaction. Centrifugation at 1000*g* for 10 min was carried out, and the optical density of the supernatant was measured at 535 nm. The degradation product of polyunsaturated fatty acids was calculated using the extinction coefficient of 1.56 10^5^ M^−1^ cm^−1^.

## 3. Results

### 3.1. The Effect of *Origanum vulgare* on Sperm Motility and Vitality

**Figure 1:**
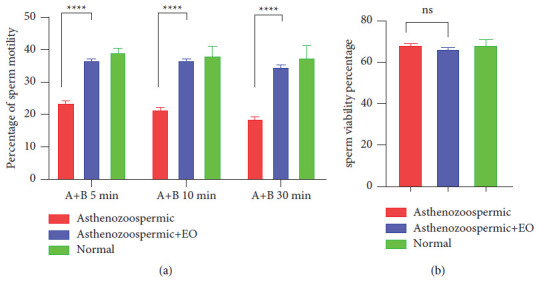
The effect of *in vitro* supplementation of EO of *O. vulgare* on progressive motility (grade “A” + “B”) (a) and vitality (b) of human spermatozoa from asthenozoospermic samples. A 0.2% (w/v) agar solution was used to dilute the EO.

The effect of supplementation of oregano essential oil on sperm motility and vitality in the studied and control groups is reported in (Figure 1). This figure shows that the incubation of human sperm with oregano OE for all holding times (5, 10, and 30 min) significantly increases the fraction of mobile sperm compared to the asthenozoospermia group. In addition, when comparing our results to the normal group, we notice a convergence of the mobility of the spermatozoa of the asthenozoospermic group treated with EO with the normozoospermic group (Figure 1(a)). However, no significant effect of oregano was observed on sperm vitality after one hour of incubation at 37°C (Figure 1(b)).

### 3.2. The Effect of *Origanum vulgare* on the DNA Fragmentation Index

**Figure 2:**
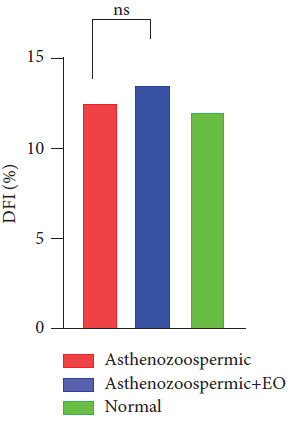
The effect of in vitro supplementation of the essential oil of oregano on DNA fragmentation of samples asthenozoospermic. A 0.2% (w/v) agar solution was used to dilute the EO. The DFI percentages of each aliquot were evaluated after the addition of the essential oil over a period of 60 minutes. The results reveal that the oil oregano has no effect on DNA fragmentation in human sperm.

The proportion of DNA-fragmented sperm in the two control and treated groups has revealed no significant difference in the proportion of DNA fragmentation: 12% and 13%, respectively (Figure 2).

### 3.3. The Effect of *Origanum vulgare* on the Antioxidant Activities of Sperm

**Figure 3:**
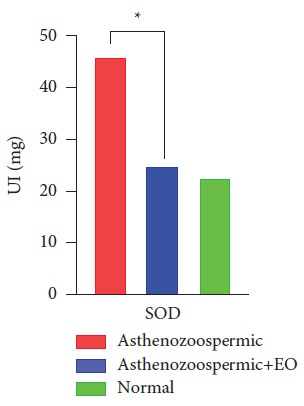
The specific activity of SOD in the group of asthenozoospermic samples, the group asthenozoospermic treated with the essential oil of oregano, and the normozoospermic group.

By analyzing the results reported in Figure 3, we found a significant increase in SOD activity in the asthenozoospermic group compared with the control group. However, the specific activity of this enzyme (SOD) has been significantly decreased after the *in vitro* supplementation of oregano EO on asthenozoospermic patients, compared to the asthenozoospermic group.

**Figure 4:**
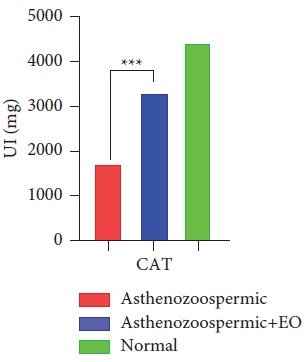
The specific activity of CAT in the group of asthenozoospermic samples, the group asthenozoospermic treated with the essential oil of oregano, and the normozoospermic group.

As illustrated in Figure 4, the normal group exhibited the best activity of catalase compared to asthenozoospermic samples. However, treating asthenozoospermic samples with oregano EO significantly increased the activity of this enzyme.

**Figure 5:**
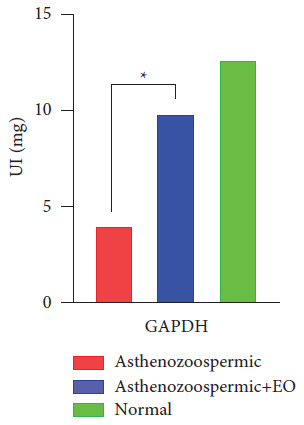
The specific activity of GAPDH in the group of asthenozoospermic samples, the group asthenozoospermic treated with the essential oil of oregano, and the normozoospermic group.

Indeed, the measurement of GAPDH reveals a significant decrease in the activity of this metabolic enzyme in asthenozoospermic samples compared with the normozoospermic group (Figure 5). However, the supplementation of oregano EO has significantly restored the activity of this enzyme.

**Figure 6:**
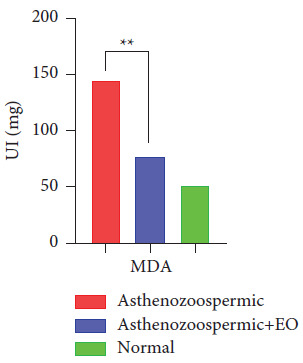
The specific activity of MDA in the group of asthenozoospermic samples, the group asthenozoospermic treated with the essential oil of oregano, and the normozoospermic group.

Likewise, the presence of the oregano EO induced a significant decrease in the MDA level in the asthenozoospermic group compared with the untreated group (Figure 6).

## 4. Discussion

The effect of *in vitro* supplementation of oregano essential oil (*O. vulgare*) on advanced parameters of the mobility of asthenozoospermic sperm has shown that the 10^−2^ dilution of this essential oil, used during 5, 10, and 30 min of incubation at 37°C and under 5% CO_2_, can increase sperm mobility by 14% compared to the control group. This allowed the samples to go from asthenozoospermic to normozoospermic samples. However, there was no discernible difference between the two DFIs when the effect of oregano oil supplementation on DNA integrity was assessed after 60 min of incubation at 37°C with 5% CO_2_. It could be argued that oregano essential oil has no effect on the DNA fragmentation index.

For biochemical analysis, the results showed a significant increase in the main enzymatic antioxidant defenses (CAT and GAPDH) as well as a decrease in SOD and MDA after supplementation with oregano essential oil. We also observed that treated groups of asthenospermia returned to normal values compared to untreated groups.

These results are in accordance with the published work of [15], which showed that the supplementation of EO of oregano has improved advanced parameters of human sperm mobility.

Other studies on rabbit semen that the spermatic parameters of the rabbit semen have improved when incubated with *O. vulgare* extract in a capacitating medium [16]. This ameliorative activity of OE may be related to the abundance of some bioactive compounds such as thymol and carvacrol, and the authors in [17] reported in their study that oral treatment of rats with different doses of thymol and carvacrol significantly increased sperm motility compared to the control group [17]. Güvenç et al. also demonstrated the protective effect of these biomolecules against lipid peroxidation [17].

However, assessing the effect of oregano oil supplementation on DNA integrity at 60 min incubation at 37°C under 5% CO_2_ did not produce a significant difference in DFI values. According to several studies, a high level of sperm fragmentation, as evidenced by the TUNEL test, appears to be associated with low fertility rates and a significantly lower pregnancy rate following IVF [18, 19]. Damage to sperm DNA can be caused by a number of conditions, including recombination deficiencies, aborted apoptosis, and oxidative stress with reactive oxygen species production (ROS) [15, 19]. The integrity of the sperm DNA and especially its mobility is an essential component of male fertility as it allows sperm to move efficiently across the female reproductive system to reach and fertilize the egg [20]. In addition, the presence of reactive oxygen species (ROS) in sperm functions is a main second messenger in the fertilization process at low levels, including capacitation, hyperactivation, acrosome reaction, and sperm fusion-oocytes [21]. Nevertheless, several studies have shown that mean levels of ROS are significantly higher in the sperm of men with abnormal parameters [22]; therefore, a balance of ROS and antioxidants is necessary for efficient sperm activity and fertilization [23].

According to several studies, the proportions of normal mobility and morphology of sperm were positively related to CAT activity [24]. This positive correlation between CAT activity and sperm motility can be explained by the importance of this enzyme in the protection of sperm from potentially toxic ROS levels [25]. Treatments with H_2_O_2_ produced an increase in MDA levels and inhibited advanced sperm motility parameters [26]. However, the incubation of human sperm with H_2_O_2_ revealed that human catalase was able to counteract the adverse effects of H_2_O_2_ on sperm motility [12]. This can be related to the role of this antioxidant enzyme in the conversion of H_2_O_2_ into molecules of water and molecular oxygen [27].

(1) $$\begin{array}{c} 2H_{2}O_{2}2H_{2}O+O_{2}\text{.} \end{array}$$

On the other hand, in human sperm, the main ROS produced is the superoxide anion, which is the subsequent conversion of the hydrogen peroxide, and that is catalyzed by superoxide dismutase (SOD) [23]. In fact, numerous studies have shown a negative correlation between SOD activities and different aspects of sperm quality examined [28, 29]. This observation can be explained by the elimination of superoxide anion which is important for the normal function of human sperm [30]. Based on this finding, it may be suggested that the increase in SOD activity and the decrease in CAT activity are related to low semen parameters.

(2) $$\begin{array}{c} 2O_{2}^{•–}+2H^{+}⟶O_{2}+H_{2}O_{2}\text{.} \end{array}$$

In this study, we reported that MDA levels in the asthenozoospermic group have increased in comparison with the normozoospermic group. Several studies in the literature have shown that lipid peroxidation impacts the concentration, motility, and morphology of sperm and is associated with poor sperm quality [31, 32] and detected that the MDA level in normospermic men was significantly lower than those in asthenoteratospermic and oligoasthenoteratospermic groups. Indeed, it is suggested that a negative correlation has been highlighted by sperm counting, studying motility, and morphology.

According to the obtained results, we detected an increase in the GAPDH activity in treated samples compared to untreated asthenozoospermic samples. 3-Phosphate dehydrogenase is a glycolytic pathway enzyme that catalyzes the conversion of 3-phosphate glycerol into 1,3-bisphosphoglyceride [33]. This glycolytic enzyme is an essential biomarker that actively contributes to the formation of cell energy in the form of ATP. This production of glycolytic ATP is necessary for sperm motility and male fertility [34].

## 5. Conclusions

This study highlighted the effects of *in vitro* supplementation with the essential oil of oregano on the advanced parameters of sperm motility and also influenced the antioxidant activity of human sperm. The results allowed us to observe an improvement in these parameters. Based on the current results, we can conclude that oregano oil could be a great tool for the management of motility dysfunction in asthenozoospermic patients.

## Data Availability

The biochemical data used to support the findings of this study are included within the article.
